# Evaluating the clinical teaching of medical imaging students at Curtin University of Technology, Australia

**DOI:** 10.2349/biij.7.3.e20

**Published:** 2011-07-01

**Authors:** HM Almohiy, R Davidson

**Affiliations:** 1 Department of Medical Imaging, King Khalid University, Abha, Saudi Arabia; 2 Department of Medical Imaging, Curtin University, Perth, Western Australia

**Keywords:** OSCE, clinical program, laboratory sessions, medical imaging education

## Abstract

**Purpose:**

To ascertain the effectiveness of the clinical, tutorial-based component of teaching and the clinical assessment method in the Bachelor of Medical Imaging Science at Curtin University of Technology (CUT), Perth, Western Australia.

**Materials and Methods:**

In mid-2006, second- and third-year students enrolled in CUT’s Medical Imaging Science degree were asked to complete a questionnaire assessing the Objective Structured Clinical Examination (OSCE) evaluation program and clinical teaching. Thirty-three of 57 students answered questions about demographics and their opinions of the laboratory sessions, clinical placements and the OSCEs.

**Results:**

Seventy-six per cent of students were satisfied with their laboratory sessions and clinical placements. Sixty-four percent of respondents indicated that the OSCE was not an objective evaluation, but 82% of students felt the OSCE was an effective test of their radiography skills and knowledge, and believed that they were able to evaluate and care for a patient during the OSCE.

**Conclusion:**

Overall, the surveyed students believed that the practical skills explored in laboratory sessions helped improve clinical training outcomes; however, only 33% of the students were satisfied that the OSCE was an appropriate assessment of their clinical training in hospitals.

## INTRODUCTION

The Curtin University of Technology (CUT) launched a Medical Imaging course in 1987, initially offering a Diploma in Medical Imaging, and later, a Bachelor’s degree course. As part of their degree, students gain practical radiography skills development during laboratory sessions held on campus, as well as during clinical placements at public hospitals and private radiology practices. Laboratory sessions commence during the students’ first year of the course. Laboratory sessions, undertaken during the students’ second and third years, are designed to equip students with appropriate clinical skills and knowledge for their clinical placements. The students are required to demonstrate effective skills in radiography in each element of practical training, so that during their final semester in their third year they should be able to perform all types of radiographic examination at beginning practitioner level on any given patient.

Boggis *et al*. [1] described the need for students to participate in different clinical settings to practise radiography, and recommended that experienced radiographers should be involved in the clinical practice as they offer an important teaching resource. Accordingly, as part of their evaluation, CUT medical imaging students undergo Objective Structured Clinical Examinations (OSCEs). The OSCEs involve several structured clinical evaluation sessions [2]. The focus of this paper is the clinical skill assessment session, performed under simulated clinical conditions [3].

Clinical teaching at CUT is comprised of supervised, lab-based practical sessions and its effectiveness has not previously been evaluated. A search of the literature revealed no previous studies looking at radiography students’ opinions of the OSCE. This paper describes an evaluation of the opinions of Bachelor of Science (Medical Imaging Science) students at CUT regarding laboratory sessions and the OSCE. A particular focus of our research was students’ perceptions about whether the practical skills explored in laboratory sessions helped to improve clinical training outcomes and whether the OSCE is an appropriate assessment of their clinical training in hospitals.

## METHODS

Students studying in the second or third year of a three-year Bachelor of Science (Medical Imaging Science) degree at CUT in 2006 participated in this study. Students were asked to complete and return a questionnaire that was designed to assess the positive and negative aspects of the OSCE. The survey was designed to undertake a comparison of the students’ views about their learning in laboratory sessions and during clinical placement. Ethical approval for the study was sought and gained from the Department of Imaging and Applied Physics at CUT. Student consent was obtained prior to participation. Part A (questions 1 to 6) of the questionnaire focused on demographics. Part B (questions 7 to 8) focused on levels of satisfaction with various aspects of the laboratory sessions and clinical placement; participants were asked to choose responses from a five-point Likert scale – ‘no satisfaction’, ‘satisfied to a small extent’, ‘satisfied to some extent’, ‘satisfied to a large extent’ or ‘satisfied to a very large extent’. Part C (questions 9 to 20) focused on the OSCE, and all questions used a five-point Likert scale – ‘strongly disagree’, ‘disagree’, ‘undecided’, ‘agree’, or ‘strongly agree’. Questionnaires were anonymous and were distributed to the students by their lecturers with pre-paid envelopes for their return.

## RESULTS

### Demographics

Fifty-seven students were given questionnaires, and 33 (57.9%) responded – 16 women and 17 men (Table 1). Most respondents were 18 to 22 years of age (57.6%), Australian-born (78.8%) and from an English-speaking background (81.8%). Seventy-six per cent described themselves as employed and two-thirds were in their third (final) year of study. The reliability for the most important questions in the questionnaire was calculated and it was found that the Cronbach alpha = 0.89.

**Table 1:** Respondent demographics (n = 33).

**Variable**	**n (%)**
Sex	
Male	16 (48.5)
Female	17 (51.5)
Age (years)	
18 to 22	19 (57.6)
23 to 26	08 (24.2)
27 to 30	02 (6.1)
>30	04 (12.1)
Country of origin	
Australia	26 (78.8)
China	2 (6.1)
India	1 (3.0)
Indonesia	1 (3.0)
Iran	2 (6.1)
United Kingdom	1 (3.0)
First language	
English	27 (81.8)
Other	6 (18.2)
Currently employed (part-time, casual or other)	25 (75.8)
Year of study	
Second year	11 (33.3)
Third year	22 (66.7)

### Laboratory sessions and clinical placements

Overall, when asked about their extent of satisfaction with their laboratory sessions and clinical placements, more students indicated they were satisfied to a large or very large extent (39.4% and 51.5% respectively) than those who responded that they were satisfied to a small extent or unsatisfied (24.2% and 15.1% respectively) (Table 2). Only one respondent was ‘not at all’ satisfied with the clinical teaching. No students responded that they were ‘not at all’ satisfied with the provision of new knowledge or skills in the laboratory sessions to help with their clinical placements. Only one student did not regard self-assessment as an important feature of their laboratory sessions (3%), and only one student did not regard self-assessment as important to the clinical placements (3%). The vast majority of students (91%) were satisfied to a large or very large extent that attending different clinical placements was important.

**Table 2:** Student level of satisfaction with various aspects of teaching*

	**Level of satisfaction**				
	**Not at all**	**To a small extent**	**To some extent**	**To a large extent**	**To a very large extent**
Laboratory sessions and clinical placement	0 (0)	8 (24.2)	12 (36.4)	11 (33.3)	2 (6.1)
Clinical teaching	1 (3.0)	4 (12.1)	11 (33.3)	14 (42.4)	3 (9.1)
Knowledge and skills to assist with clinical placement	0 (0)	7 (21.2)	13 (39.4)	11 (33.3)	2 (6.1)
Applicability of laboratory sessions with the OSCE	0 (0)	6 (18.2)	13 (39.4)	9 (27.3)	5 (15.2)
Self-assessment in relation to laboratory sessions	1 (3.0)	4 (12.1)	14 (42.4)	13 (39.4)	1 (3.0)
Self-assessment in relation to clinical placements	1 (3.0)	2 (6.1)	11 (33.3)	16 (48.5)	3 (9.1)
Importance of attending different clinical placements	0 (0)	0 (0)	3 (9.1)	8 (24.2)	22 (66.7)

* n = 33, percentages in parentheses.

When asked how the laboratory sessions and clinical placements could be improved, respondents suggested that the number of laboratory sessions and the time spent in them should be increased; that the structure of laboratory sessions could be improved; that extra equipment and more time spent using the equipment was required; and that the lecturer should give the laboratory session instead of an instructor. In addition, students wanted to see the link between the laboratory sessions and the OSCE.

### Objective Structured Clinical Examinations (OSCE)

Sixty-seven per cent of respondents agreed that the OSCE was an effective test of their radiography skills and knowledge, and 55% of respondents believed they were able to evaluate and care for a patient during the OSCE (Table 3). Seventy-nine per cent of respondents agreed that it would be useful to receive feedback during their OSCE. Forty-two per cent of respondents were undecided as to whether self-assessment should be included as part of the OSCE, but 39.4% of respondents agreed with it. Students were divided with respect to the use of video recording of the OSCE as part of self-assessment (39.2% disagreed or strongly disagreed; 39.4% agreed or strongly agreed), but were overwhelmingly in favour of using video as an aid to examiner assessment (63.6% agreed or strongly agreed). Only two respondents (6.1%) were opposed to the idea of developing their own performance criteria for the OSCE; 48% supported the idea of more than one OSCE examiner. The majority of respondents (66.7%) agreed or strongly agreed that the resources to assist them with their OSCE preparation were helpful; however, 63.7% of respondents disagreed or strongly disagreed that the OSCE is more objective than a clinical evaluation. In addition, 45% of respondents disagreed or strongly disagreed that they were satisfied with the OSCE, compared to 33% who agreed or strongly agreed.

**Table 3:** Student opinions on effectiveness of OSCE test.

	**Response**				
	**Strongly Disagree**	**Disagree**	**Undecided**	**Agree**	**Strongly agree**
The OSCE tests my performance at demonstrating the required skills and knowledge I was expected to know.	2 (6.1)	4 (12.1)	5 (15.1)	20 (60.6)	2 (6.1)
I was able to plan, implement and evaluate care of the patient during the OSCE.	3 (9.1)	4 (12.1)	8 (24.2)	16 (48.5)	2 (6.1)
I would find it useful to receive feedback during the OSCE.	1 (3.0)	3 (9.1)	3 (9.1)	12 (36.4)	14 (42.4)
I would like to have self-assessment introduced as part of the OSCE.	3 (9.1)	3 (9.1)	14 (42.4)	12 (36.4)	1 (3.0)
If self-assessment was a part of the OSCE I would agree to be videotaped to help with this.	7 (21.2)	6 (18.2)	7 (21.2)	10 (30.3)	3 (9.1)
If a video of me was taken during the OSCE I would agree that the examiners could use this to reappraise my performance.	4 (12.1)	1 (3.0)	7 (21.2)	20 (60.6)	1 (3.0)
I would agree to develop my own performance criteria for the OSCE.	0 (0)	2 (6.1)	11 (33.3)	20 (60.6)	0 (0)
I would like to have more than one examiner present during the OSCE.	2 (6.1)	7 (21.2)	8 (24.2)	10 (30.3)	6 (18.2)
The radiography texts and other resources were helpful in preparation for the OSCE.	2 (6.1)	4 (12.1)	5 (15.1)	20 (60.6)	2 (6.1)
The OSCE is a more objective examination of my clinical skills rather than being evaluated at the clinical setting.	12 (36.4)	9 (27.3)	3 (9.1)	5 (15.2)	4 (12.1)
Overall, I am satisfied with the OSCE examination.	5 (15.1)	10 (30.3)	7 (21.2)	10 (30.3)	1 (3.0)

n = 33, the numbers in parentheses are the percentages.

When asked how the OSCE could be improved, respondents suggested that the evaluation of their clinical skills would be better performed during clinical placements rather than during the OSCE, and that practising radiographers, rather than lecturers, may be better placed to assess students during the OSCE. Other respondents stated that the OSCE was not a fair evaluation and that better equipment was required during the OSCE for more realistic simulations.

## DISCUSSION

In this study, medical imaging students mostly agreed that the laboratory sessions were important for developing practical skills and improving clinical training outcomes. Students were generally satisfied with clinical and laboratory teaching, noted the value of self-assessment during these sessions, and agreed that attendance at different clinical settings was essential for their training. Some students suggested that additional laboratory sessions could be scheduled to provide more hands-on time with the equipment. Our findings were consistent with those of a literature review of radiography education, which described the laboratory as an ideal environment for developing knowledge, understanding and skills [4]. Laboratory sessions provide students with the opportunity to practise radiography skills in a structured, predictable and safe learning environment, so that during clinical placement students can further develop their skills in situations that are unable to be simulated in the laboratory sessions.

Forty-five per cent of respondents were not satisfied with OSCE as an assessment of their clinical competence. These respondents recommended assessment during clinical placement rather than during OCSE. They also recommended the use of radiographers, rather than lecturers, as evaluators during the OSCE to ensure that it is conducted realistically by people who practise radiography on a regular basis. Students must be able to identify the links between laboratory sessions, clinical placements and the OSCE. A majority of students surveyed (63.7%) did not believe that the OSCE was a more objective means of evaluation than being evaluated in the clinical setting.

These results reaffirmed the students’ view that the OSCE lacks objectivity compared with clinical evaluation during clinical placement, in contrast to research that describes the advantages of the OSCE as an evaluation tool [5]. Specific reasons for this view were not identified in this study. Improving students’ perceptions about the OSCE may be possible by trialing measures to ensure that the evaluation is fair, such as the reappraisal of a video recording of the student’s performance during the OSCE; the presence of more than one examiner to ensure objectivity; and student development of the OSCE criteria. Double marking could be integrated into the OSCE to ensure reliability [6] and a second examiner could appraise the student’s performance during the OSCE or in a video recording of the student. Sixty per cent of students supported the use of a video recording of their performance; however, the logistics of time and cost would need to be examined to determine the viability of this method.

Nearly 80% of students supported the idea of receiving feedback during the OSCE. Students may become stressed under OSCE conditions and feedback provided by OSCE examiners during the evaluation may reassure students. In one study [6], feedback during the OSCE was positively received and did not alter test reliability. Examiner input during the OSCE is an excellent opportunity for clinical teaching and provides students with a chance to self-evaluate. Furthermore, the OSCE can identify problem areas within the curriculum so that adjustments can be made.

The limitations of this study included the use of an unvalidated questionnaire; however, there is no standardised instrument specifically designed to test students’ perceptions of the OSCE. The response rate of 57.9% is a further limitation, as the responses of the non-participating students may have been different to those of the participants. This may be due to the administration of the exam, as the students may feel that the proctor is not experienced and/or not evaluating their work correctly. One of the questions compared the OSCE and the clinical examination as objective evaluations, whereby students expressed conflicting perceptions between these two evaluations.

## CONCLUSION

The results of this study suggest that second- and third-year students enrolled in CUT’s Bachelor of Science (Medical Imaging Science) believe that the practical skills explored in laboratory sessions help to improve clinical training outcomes. However, most students were not satisfied that the OSCE is an appropriate assessment of their clinical training in hospitals. This is the first study to provide data on medical imaging students in Australia, and as such, it represents valuable feedback on students’ perceptions of the importance and effectiveness of current teaching practices.
